# Exercise capacity after mechanical circulatory support compared to heart transplant in advanced heart failure

**DOI:** 10.1016/j.jhlto.2026.100484

**Published:** 2026-01-08

**Authors:** Hui Zhen Lo, Louise Fuller, Peter Bergin, Caitlin Cheshire, Sarah Gutman, James Hare, David M. Kaye, Hitesh Patel, Janelle Mclean, Julia Rix, Andrew J. Taylor

**Affiliations:** a Peninsula Health, Frankston, Victoria, Australia; b Department of Physiotherapy, The Alfred, Melbourne, Victoria, Australia; c Department of Cardiology, The Alfred, Melbourne, Victoria, Australia; d Baker Heart & Diabetes Institute, Melbourne, Victoria, Australia; e Faculty of Medicine, Nursing and Health Sciences, Monash University, Melbourne, Victoria, Australia; f Department of Cardiology, Royal Melbourne Hospital, Melbourne, Victoria, Australia

**Keywords:** orthotopic heart transplant, mechanical circulatory support, ventricular assist device, heart failure, exercise capacity

## Abstract

**Background:**

In patients with advanced heart failure, it is assumed that orthotopic heart transplant (OHTx) results in greater exercise capacity compared to ventricular assist device (VAD), however this has not been formally examined. In this study, we evaluated the exercise capacity of patients following VAD and OHTx after a structured outpatient rehabilitation program.

**Methods:**

We performed a retrospective single-center cohort study of patients undergoing OHTx or VAD at a tertiary centre from January 2022 to January 2024. Comorbidities and the option of either intervention was used as inputs in a multivariate linear regression model for prediction of 3-month 6-minute walk test (6MWT). Major factors identified were added as covariates in the combined cohort to determine predictors of 6MWT.

**Results:**

A total of 78 patients were included. The maximal recorded 6MWT was similar between both cohorts (median 6MWT OHTx: 534.0 (478.8-600.3)m; VAD: 608.5 (487.0-643.5)m, *p*=0.30). Gender was a significant predictor of 6MWT in the OHTx cohort (β=-69.1, *p*<0.02) with a strong trend in the VAD cohort (β=-199, *p*<0.09). VAD was not predictive of 3-month 6MWT (*p*=0.99) in OHTx patients bridged with VAD. VAD or OHTx was also not predictive of 6MWT (*p*=0.88) while total length of stay was a significant predictor of 6MWT (*p*<0.0001) in the combined cohort.

**Conclusions:**

In patients with advanced heart failure, VAD and OHTx are associated with a similar level of exercise capacity and does not impact exercise capacity in patients undergoing OHTx who are bridged with a VAD. In patients with OHTx, gender was the strongest predictor of exercise capacity.

## Background

Heart failure is a common condition worldwide. In Australia, it is estimated that patients aged 65 years and above make up two-thirds of the total number of adults with heart failure.^1^ In the United States, heart failure treatment/care is a major health care cost, and it is predicted that the cost of care will reach $69.8 billion by 2030.^2^ Even with the significant expenditure spent on the management of heart failure patients, heart failure and cardiomyopathy still accounted for 15% of total deaths in 2021.^1^

Currently, the treatment of heart failure consists of nonpharmacological and pharmacological interventions, as well as devices and interventional therapies. In the short-term, patients are advised to exercise regularly and restrict sodium along with compliance with guideline–directed medical therapy.^3^ Interventional therapies include implantable cardiac defibrillators and cardiac resynchronization therapies.^3^ These management options have significantly improved both mortality and morbidity of patients. However, many still ultimately succumb to this clinical syndrome, and mechanical circulatory support (MCS) and orthotopic heart transplant (OHTx) are the only viable options in prolonging the lives of patients with advanced heart failure.^4^

The ventricular assist device (VAD) is a form of MCS that mimics the left ventricle [left ventricular assist device (LVAD)], right ventricle [right ventricular assist device (RVAD)], or both ventricles [biventricular assist device (BiVAD)]. The most common device inserted is the LVAD and it accounts for more than 2500 implants a year in the United States.^5^ LVAD is a mechanical pump that substitutes the function of the left ventricle and is typically used as a bridge to cardiac transplant (BTT). More recently, LVAD has been used for destination therapy (DT) in those deemed unsuitable for an OHTx.^3^, ^6^, ^7^

The median survival after cardiac transplant in an adult is more than 12 years^8^ which is significantly longer than that following DT in early VAD trials.^9^, ^10^ However, this gap appears to be narrowing with the recent MOMENTUM 3 trial in 2022 also showing a 58.4% 5-year survival. One important risk factor for 1-year post-transplant mortality is having an LVAD for more than 6 months.^7^ Despite this, many patients tend to be on LVAD as a BTT due to the scarcity of heart donors. In Australia and New Zealand, the average wait time for a cardiac transplant is 158 ± 216 days.^11^

While the gap in projected survival between VAD and OHTx continues to narrow, OHTx is still considered the therapy of choice in those patients for whom this therapy is available. A significant factor beyond the survival benefit compared to VAD is the assumption that OHTx also results in greater exercise capacity. Of note, previous studies comparing VAD and OHTx were done in 2011 and 2014.^12^, ^13^ Jakovljevic et al studied patients on HeartWare, which has since been discontinued.^13^ While Kugler et al studied patients on HeartMate II, the Momentum 3 trial has shown a significant mortality benefit for HeartMate III, which has since been the VAD device of choice and also the VAD device used in our MCS center.^12^ Hence, in this study, we provide an up-to-date evaluation of the exercise capacity of patients following VAD and OHTx after a structured outpatient rehabilitation program.

## Methods

### Data sources and sample

Data were obtained from a major OHTx and MCS center, from January 2022 through January 2024. This retrospective single-center study included any patient in the OHTx or mechanical support population at our center. Patients who were undergoing VAD exchange or already had an OHTx were excluded. Baseline characteristics, including age, gender, comorbidities (atrial fibrillation [AF], diabetes mellitus [DM], chronic kidney disease [CKD], chronic lung disease [CLD]), length of stay in the intensive care unit (ICU), and total length of stay postoperatively, were collected. Ethics approval was granted before the commencement of data collection.

### Outcome measures

All patients underwent a standardized exercise program as an outpatient for a minimum of 3 months following surgery. The program consisted of cardio-vascular training on a treadmill and cycle ergometer and upper and lower limb muscle strength training. Exercises were progressed, and the program was supervised thrice weekly for both patients with VAD and OHTx. The study end-point was the 6-minute walk test (6MWT) recorded at 3 months postsurgery and the maximum 6MWT postsurgery. The 3-month 6MWT was chosen to allow time for rehabilitation and physiotherapy. For patients who had continual monthly follow-up physiotherapy, including a 6MWT at the hospital, the best 6MWT was obtained, which is defined as the longest distance walked in a single 6MWT. This could be any 6MWT from the first-month postsurgery to 35 months postsurgery.

### 6-minute walk test procedure

The 6MWT was performed at the hospital with an experienced physiotherapist. Before each 6MWT, the patient’s baseline blood pressure, heart rate (HR), oxygen saturation (SpO_2_), and dyspnea status using the Borg scale were recorded. An oximeter was attached to the patient to monitor SpO_2_ and HR throughout the test. Patients walk independently on flat ground along a 30-m track according to a standard protocol.^14^ After 6 minutes, the distance walked, HR, SpO_2_, HR, and dyspnea status using the Borg scale^15^ will be recorded.

### Data analysis

Categorical variables are presented as frequencies and proportions. Continuous variables are presented as mean and standard deviation or median (interquartile range) as appropriate. Multivariate linear regression was performed to examine the relationship between 6MWT of patients in the VAD or OHTx cohort, and their comorbidities using STATA v.17. The regression coefficient (β) was used to assess the explanatory power of the 6MWT and assumptions of multiple linear regression were maintained. An analysis of covariance was subsequently performed to evaluate the relationship between 6MWT of patients in the combined population (VAD and OHTx) and chosen comorbidities using STATA v.17 (StataCorp LLC).

## Results

**Figure 1:**
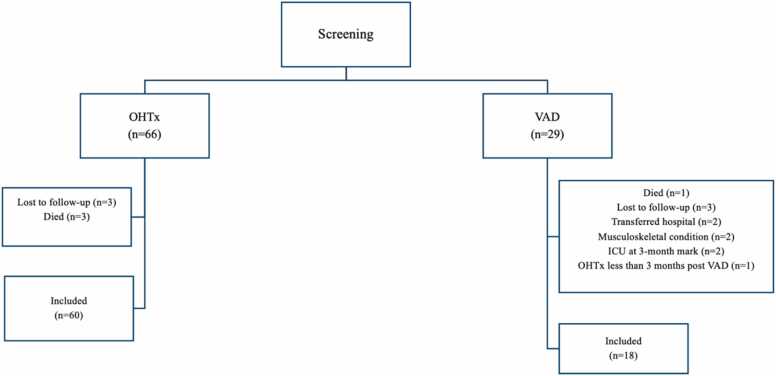
Patient flow consort diagram. ICU, intensive care unit; OHTx, orthotopic heart transplant; VAD, ventricular assist device.

**Figure 2:**
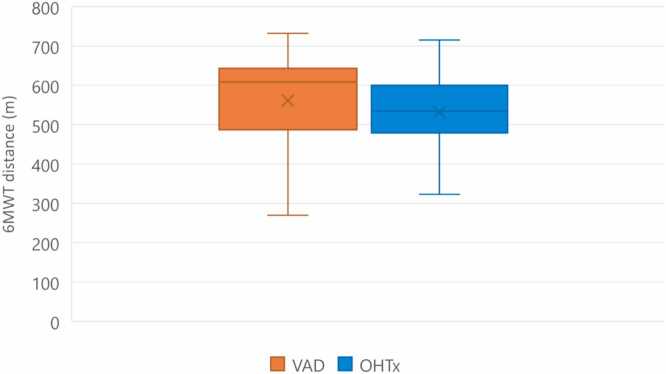
VAD vs OHTx maximal 6MWT distance. 6MWT, 6-minute walk test; m, meters; OHTx, orthotopic heart transplant; VAD, ventricular assist device.

**Figure 3:**
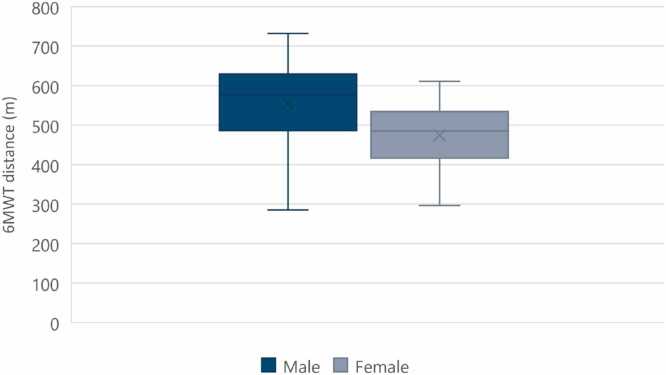
Male vs female (VAD and OHTx) maximal 6MWT distance. 6MWT, 6-minute walk test; m, meters; OHTx, orthotopic heart transplant; VAD, ventricular assist device.

A total of 29 patients with VAD and 66 patients with OHTx were screened for inclusion. Of these, a total of 78 patients (60 OHTx and 18 VAD) met the inclusion criteria after accounting for 3 patients who were lost to follow-up and 3 patients who died in the OHTx population as well as 3 patients who were lost to follow-up, 1 patient who died, 2 patients who had transfer of care to another hospital, 2 patients who had musculoskeletal conditions affecting ability to proceed with 6MWT, 2 patients who were still in ICU at the 3-month mark, and 1 patient who had an OHTx less than 3 months post VAD in the VAD population (Figure 1). Characteristics of patients were included in Table 1. The majority of patients in the VAD population were males (94.4%), median height of 175.8 (172-178.8) m, median weight of 82.8 (70.6-99.3) kg, median age of VAD insertion was 50.0 (44.3-56.8) years, 38.9% had AF/atrial flutter, 22.2% had DM, 38.9% had chronic kidney disease, 5.6% had CLD, median total length of stay in the hospital was 43.0 (33.5-84) days, median length of stay in the ICU was 23.0 (14.0-41.0) days, and median maximal 6MWT was 608.5 (487.0-643.5) m (Figure 2). The majority of patients in the OHTx population were also males (75.0%), median height of 173.5 (163.8-180.3) m, median weight of 81.3 (65.7-91.1) kg, median age at transplantation of 52.0 (44.8-59.0) years, 38.3% had AF/atrial flutter, 21.7% had DM, 23.3% had chronic kidney disease, 10.0% had CLD, 35.0% had MCS before OHTx, median total length of stay in the hospital was 24.0 (16.0-32.3) days, median length of stay in the ICU was 10.0 (5.8-15.3) days, and median maximal 6MWT was 534.0 (478.8-600.3) m (Figure 2). Comparing all males to females in both the VAD and OHTx population, the median maximal 6MWT distance for males vs females was 576.5 (486.5-629.8) m and 485.0 (416.0-534.5) m, respectively (Figure 3).

**Table 1:** Characteristics of Patients With Ventricular Assist Device or Orthotopic Heart Transplant

Patient characteristics	Patients with VAD, *n* = 18	Patients with OHTx, *n* = 60
Male sex (%)	17 (94.4)	45 (75.0)
Female gender (%)	1 (5.6)	15 (25.0)
Height (m)	175.8 (172-178.8)	173.5 (163.8-180.3)
Weight (kg)	82.8 (70.6-99.3)	81.3 (65.7-91.1)
Median age at insertion, years (95% CI)	50.0 (44.3-56.8)	52.0 (44.8-59.0)
Median total length of stay, days (95% CI)	43.0 (33.5-84)	24.0 (16.0-32.3)
Median length of ICU stay, days (95% CI)	23.0 (14.0-41.0)	10.0 (5.8-15.3)
Median maximal 6MWT, m (95% CI)	608.5 (487.0-643.5)	534.0 (478.8-600.3)
Atrial fibrillation/atrial flutter (%)	7 (38.9)	23 (38.3)
Diabetes mellitus (%)	4 (22.2)	13 (21.7)
Chronic kidney disease (%)	7 (38.9)	14 (23.3)
Chronic lung disease (%)	1 (5.6)	6 (10.0)
Prior MCS (%)	NA	21 (35.0)

Abbreviations: 6MWT, 6-minute walk test; CI, confidence interval; ICU, intensive care unit; MCS, mechanical circulatory system; NA, not applicable; OHTx, orthotopic heart transplant; VAD, ventricular assist device.

### Predictors of exercise capacity in patients with VAD

The multivariate linear regression model was created to predict 6MWT for patients with VAD from gender, age, glomerular filtration rate (GFR), AF, DM, CLD, total length of stay in hospital, and length of stay in the ICU. For patients with VAD, the model significantly predicted 6MWT (R^2^ = 0.9, *p* < 0.001). Due to the small sample size of 18 patients, no comorbidities were statistically significant predictors of 6MWT, although there was a strong trend for female gender (β = −199, *p* < 0.09). The correlations between 6MWT and the comorbidities are included in Table 2.

**Table 2:** Multiple Linear Regression of 3-month 6MWT in Ventricular Assist Device Patients Only Stratified by Categorical Study-level Characteristics Using the Random Effect Model

Variable	Coefficient	*p*-value
Gender	−198.9228	0.092
Age	−2.224361	0.242
GFR	−0.3837358	0.613
AF	−34.80757	0.368
DM	−111.9978	0.127
CLD	−25.43373	0.786
Total LOS	−1.328879	0.338
ICU LOS	−1.532244	0.289
Constant	582.2334	0.001

Abbreviations: 6MWT, 6-minute walk test; AF, atrial fibrillation; CLD, chronic lung disease; DM, diabetes mellitus; GFR, glomerular filtration rate; ICU, intensive care unit; LOS, length of stay.

### Predictors of exercise capacity in patients with OHTx

The regression model was used to predict 6MWT for patients with OHTx from gender, age, GFR, AF, DM, CLD, total length of stay in hospital, length of stay in the ICU, and prior MCS. For patients with OHTx, the model significantly predicted 6MWT (R^2^ = 0.3, *p* < 0.05). Only gender remained a significant predictor of 6MWT in OHTx population (β = −69.1, *p* < 0.02). In patients undergoing OHTx with a prior VAD implantation, VAD was not predictive of 6MWT following OHTx (*p* = 0.99). The correlations between 6MWT and the comorbidities are included in Table 3.

**Table 3:** Multiple Linear Regression of 3-month 6MWT Orthotopic Heart Transplant Patients Only Stratified by Categorical Study-level Characteristics Using the Random Effect Model

Variable	Coefficient	*p*-value
Gender	−69.06196	0.024
Age	−2.043352	0.122
GFR	−0.3893931	0.619
AF	19.93434	0.404
DM	−33.95422	0.297
CLD	26.70496	0.475
Total LOS	−0.602667	0.077
ICU LOS	−1.452574	0.282
Prior MCS	0.2241036	0.993
Constant	642.7034	0.000

Abbreviations: 6MWT, 6-minute walk test; AF, atrial fibrillation; CLD, chronic lung disease; DM, diabetes mellitus; GFR, glomerular filtration rate; ICU, intensive care unit; LOS, length of stay; MCS, mechanical circulatory system

### Predictors of exercise capacity in all patients

Given the risk of overfitting, an analysis of covariance was utilized to predict 6MWT for the combined patients with VAD and OHTx from gender, age, and either intervention of VAD or OHTx. These variables were chosen given that they were significantly associated or had strong trends to shorter 6MWT in the VAD or OHTx multivariate linear regression analysis. For all patients, total length of stay was significantly associated with 6MWT (*p* < 0.0001). In contrast, the option of VAD or OHTx was not predictive of 6MWT (*p* = 0.88). The correlations between 6MWT and the comorbidities are included in Table 4.

**Table 4:** Analysis of Covariance of 3-month 6MWT in Patients With Ventricular Assist Device or Orthotopic Heart Transplant Stratified by Categorical Study-level Characteristics

Variable	Partial SS	MS	F ratio	*p*-value
VAD or OHTx	657,604.671	16,861.6582	2.72	0.8856
Age	130.317357	130.317357	0.02	0.2336
Gender	3,556.6267	3,556.6267	0.57	0.4538
Total LOS	127,691.505	127,691.505	20.57	0.0001

Abbreviations: 6MWT, 6-minute walk test; LOS, length of stay; MS, mean squares; OHTx, orthotopic heart transplant; SS, sums of squares; VAD, ventricular assist device.

## Discussion

In this retrospective study, we demonstrated that, in patients with advanced heart failure, patients with VAD had similar exercise capacity to patients with OHTx following completion of cardiac rehabilitation. In patients with OHTx, gender was the major predictive factor for 6MWT, with a strong trend in the VAD cohort. Bridging VAD, however, did not affect 6MWT in patients with OHTx. Other factors, such as total length of stay, were predictive of 6MWT in the combined VAD and OHTx population.

The maximal recorded 6MWT was similar between patients with VAD and OHTx, which provides critical evidence to debunk the assumption that patients with OHTx have greater exercise capacity than patients with VAD. This result was also observed in a study in 2021 looking at the efficacy of cardiac rehabilitation in patients with VAD in comparison to patients with OHTx.^16^ However, it is worth noting that patients with VAD tend to be sicker compared to their OHTx counterparts, as shown in the longer median total length of stay (43.0 vs 24.0 days) and median ICU stay (23.0 vs 10.0 days) in patients with VAD. While no preoperative 6MWT was obtained, we can extrapolate from the baseline data that patients with VAD would have lower baseline 6MWT compared to OHTx. Hence, while the absolute maximal recorded 6MWT had no significant difference between patients with VAD and OHTx, patients with VAD would have had a larger improvement from their baseline 6MWT. 6MWT has been proven to be clinically significant in terms of providing a reliable quantification of functional capacity.^17^ This shows that cardiac rehabilitation involving a specific exercise program, such as aerobic and strength training, can help patients with VAD and OHTx improve their functional capacity, which translates to improvements in exercise capacity and activities of daily living. However, while exercise capacity can be improved with cardiac rehabilitation, incomplete adherence to regular sessions is associated with increased postoperative complications and major bleeding, signifying long-term impacts on such complications.^18^

The most significant finding in this study is that gender was the strongest predictor for exercise capacity after OHTx, as well as a strong trend after VAD. It has historically been known that the male gender has a physiological advantage compared to the female gender. While there are many factors that can contribute to this, including muscle strength and cardiac output, one of the main reasons is due to the difference in the maximum whole-body oxygen consumption (VO_2max_) between males and females.^19^ Even though cardiopulmonary exercise testing was not one of the outcome measures of this study, literature has revealed that the fittest female athlete would have approximately 10% lower VO_2max_ in comparison to the fittest male athlete.^19^ VO_2max_ is calculated by multiplying the maximum cardiac output with the maximum arterio-venous oxygen content difference.^20^ Hence, larger VO_2max_ and cardiac output contribute to the greater exercise capacity in males.

Our analysis also showed that having a VAD before OHTx did not affect 6MWT following transplantation. Extrapolating from this, we can expect a similar level of exercise capacity for any given patient with either intervention, with VAD being a BTT or DT. Thus, quality of life is expected to be similar in patients with either intervention as well. Additionally, a recent meta-analysis also showed that patients with OHTx and LVAD had no difference in 1-year mortality.^21^ Hence, these suggest that VAD could potentially be comparable to OHTx as a DT due to the similar 6MWT results and mortality rates.

A further analysis was also done in the combined VAD and OHTx population, comparing only a limited number of key variables. This secondary analysis was performed to mitigate the risk of overfitting in the combined population, given that only a very small number of patients with VAD were included in this study. Only age, gender, total length of stay, and either intervention of VAD or OHTx were the factors used in the analysis. This analysis demonstrated that only the total length of stay was significantly associated with 6MWT in the combined population and having a VAD or OHTx was not associated with 6WMT. Longer lengths of hospital stay in patients with VAD or OHTx are associated with worse outcomes, mainly due to the baseline condition of the patient.^22^, ^23^ Patients with VAD tend to spend a longer time in hospital as they are sicker compared to their OHTx counterparts. For instance, a 2019 study comparing VAD vs OHTx outcomes reported that 46% of the LVAD population had an INTERMACS profile of 1 or 2.^24^ Thus, it is nearly impossible to put these patients on the OHTx waitlist for a suitable donor, hence most of these patients will have LVAD as a BTT, thus requiring more time in rehabilitation to get to their maximal exercise capacity.

While the multivariate model was strongly predictive of 6MWT results in the VAD subgroup (with an R^2^ value of 0.9), for the OHTx subgroup, the model was only weakly predictive of 6MWT (with an R^2^ value of 0.3). This suggests that additional factors related to OHTx, such as the quality of organ implanted, ischemic time, and closeness of immunological match, are likely to affect the exercise capacity of these patients. While research has not been done to investigate the factors affecting exercise capacity in patients with OHTx, substantial research has been conducted to look at factors affecting mortality in patients with OHTx. Donor risk factors associated with post-OHTx mortality include donor-heart ischemic time, mismatch of sex, comorbidities such as hepatitis C, insulin-dependent DM, and age.^25^ Specifically, increased age of donor was associated with increased mortality in OHTx recipients.^26^, ^27^ Undermatching the size of the donor heart to the recipient was also associated with higher mortality.^28^ The strong correlation between the quality and match of donor to recipient with mortality would be similar to the correlation with exercise capacity.

To our knowledge, this is the first paper comparing exercise capacity between patients with VAD and OHTx. However, this study does not come without limitations. First, this is a retrospective, nonrandomized study with data that were limited to patients in a single tertiary center. This limits external validity, given that other hospitals may have differing clinical practices, which affects the outcomes of patients who had a VAD or OHTx. Future prospective studies would need to involve multicenters to validate our findings. It would also be valuable to compare the different cardiac rehabilitation programs in various sites to further analyze the optimal cardiac rehabilitation program for patients who have undergone a significant cardiac surgery. Our small sample size of 78 patients also limits the statistical power of the analysis, particularly in patients with VAD. There was also a risk of overfitting in a multivariate linear regression of the combined population. To mitigate this, we performed a secondary analysis using analysis of covariance with only a few key factors. This allowed for a good balance between identifying potential factors associated with 6MWT while also ensuring that appropriate statistical analysis was performed. Furthermore, we only looked at the 6MWT in all patients at 3-months postsurgery as this was the typical period of intensive rehabilitation. A longer follow-up period would be beneficial in looking at the long-term changes in exercise capacity and associated factors contributing to improvement or decline in function. Lastly, it is important to note that patients with OHTx are required to be on immunosuppressive drugs early post-OHTx to prevent rejection, including corticosteroids and calcineurin inhibitors. While immunosuppression is vital in this patient group, it also results in functional impairments. Patients can experience increased muscle atrophy and weakness, pulmonary and vascular dysfunction, as well as cardiac hypertrophy and fibrosis, which all result in poorer exercise capacity.^29^, ^30^ As our paper only looked at short-term exercise capacity, these patients were early postsurgery and were on many immunosuppression medications. It is important for future studies to look at long-term exercise capacity, especially after steroid weaning so that we can compare the long-term outcomes of patients with VAD and OHTx.

## Conclusion

In patients with advanced heart failure, when compared to OHTx, VAD is associated with a similar level of exercise capacity and does not impact exercise capacity in patients undergoing OHTx who are bridged with an LVAD. In patients with OHTx, gender was the strongest predictor of exercise capacity, with a significantly lower 6MWT seen in females. Future research could work on larger prospective cohorts to corroborate our findings of exercise capacity in patients with VAD and OHTx.

## Author Contributions

**Hui Zhen Lo:** Conceptualization, Methodology, Validation, Investigation, Data curation, Writing – original draft, Writing – review & editing, Visualization. **Louise Fuller:** Methodology, Validation, Investigation, Resources, Data curation, Writing – review & editing. **Peter Bergin:** Investigation, Resources, Data curation, Writing – review & editing. **Caitlin Cheshire:** Investigation, Resources, Data curation, Writing – review & editing. **Sarah Gutman:** Investigation, Resources, Data curation, Writing – review & editing. **James Hare:** Investigation, Resources, Data curation, Writing – review & editing. **David M. Kaye:** Investigation, Resources, Data curation, Writing – review & editing. **Hitesh Patel:** Investigation, Resources, Data curation, Writing – review & editing. **Janelle Mclean:** Investigation, Resources, Data curation, Writing – review & editing. **Julia Rix:** Investigation, Resources, Data curation, Writing – review & editing. **Andrew J. Taylor:** Conceptualization, Methodology, Software, Validation, Formal analysis, Resources, Data curation, Writing – review & editing, Supervision, Project administration.

## Statement of Ethics

Ethical approval was obtained from the institutional Ethics Committee (549/23) before commencement of the project.

## Sources of Funding

This work was supported by the 10.13039/501100000925NHMRC Investigator Grant.

## Declaration of Competing Interest

The authors declare that they have no known competing financial interests or personal relationships that could have appeared to influence the work reported in this paper.
